# Improving Quality of Care for Patients With Attention-Deficit/Hyperactivity Disorder in an Early Intervention for Psychosis Service

**DOI:** 10.1192/bjo.2024.377

**Published:** 2024-08-01

**Authors:** Masab Hanan, Marlene Kelbrick

**Affiliations:** NHFT, Northampton/Kettering, United Kingdom

## Abstract

**Aims:**

Attention-deficit/hyperactivity disorder (ADHD) is frequently associated with other psychiatric conditions, including psychotic disorders. There is evidence for shared genetic susceptibility to both conditions (Hamshere ML, Stergiakouli E, Langley K, et al. Shared polygenic contribution between childhood attention-deficit hyperactivity disorder and adult schizophrenia. Br J Psychiatry. 2013;203(2):107–111) and first-degree relatives of people with ADHD have twice the risk of having schizophrenia compared with healthy controls (Larsson H, Rydén E, Boman M, et al. Risk of bipolar disorder and schizophrenia in relatives of people with attention-deficit hyperactivity disorder. Br J Psychiatry. 2013;203(2):103–106)

There are treatment challenges, and monitoring of physical health has differences with the Early Intervention Psychosis standards following antipsychotic monitoring.

Combined outcomes (symptomatic and functional recovery) may be worse unless addressing ADHD in addition to psychosis, and addressing substance use is important.

**Primary objective/aim:**

To create an integrated pathway through a clinical practice guideline for patients with ADHD in early intervention for psychosis service.

**Secondary objectives/aims:**

Identify the prevalence of ADHD in EIP service.

Little is known about the prevalence of comorbid ADHD in adults under the care of early intervention for psychosis (EIP) services in the UK. A previous audit and quality improvement project examining all (adolescent and adult) patients (age 14–65 years) conducted in October 2022 showed a prevalence of 3.6% comorbid diagnosed ADHD in our EIP service.

**Methods:**

Audit tools to identify the prevalence of ADHD in patients with First episode psychosis. Re-audit to monitor the the trajectory of the cases.

Measurable: Using EIP caseload cross-sectional data, data of monitoring and management from electronic patient notes system (SystmOne).

A clinical pathway was developed to integrate the diagnostic and treatment pathway to manage ADHD in an early intervention psychosis service.

Service evaluation

We identified all adult patients (between the ages of 18 and 65 years) on the caseload of the EIP service in Northamptonshire Healthcare NHS Foundation Trust, dated 5 September 2023. Data collected included age, gender, ethnicity, primary psychosis diagnosis, psychotropic and ADHD medication prescribed and risk profile that included a history or presence of substance use. Data was anonymised.

Quality improvement

Following from a previous ADHD service evaluation and QIP in EIP (2022) we identified further problem areas that included significant delays in suspected comorbid ADHD in adult patients under EIP care, as well as challenges for those already referred and attempts made by the adult ADHD service for screening and assessment (poor engagement), as well as challenges to patient functional recovery and outcomes in those with comorbid diagnosed and suspected ADHD and psychosis.

**Results:**

174 of 183 (95%) patients on the caseload at the time of the study review date were adults. Of the adult patients, 5 of 174 (2.9%) patients had a known diagnosis of ADHD, all dating back to childhood/adolescence. An additional 4 patients had been referred for ADHD assessment.

**Conclusion:**

There are treatment challenges, and monitoring of physical health has differences with the Early Intervention Psychosis (EIP) standards following antipsychotic monitoring, hence why we have implemented in a lead-up ADHD in EIP Quality Improvement Project a NICE concordant care plan that includes physical health monitoring content and frequency.

Combined outcomes (symptomatic and functional recovery) may be worse unless ADHD related symptoms and functional impairment are addressed, in addition to psychosis, and addressing substance use is also important, given that in people with substance use disorder, the prevalence of ADHD is estimated to be as high as 21% (Rohner et al. 2023), with a lifetime prevalence of drug use disorder 27.7% (Anker et al. 2020). Of interest, in our cohort, 3/5 (60%) patients had a history of substance use (40% drug-induced psychosis diagnosis).

Our study patient profile included all young Caucasian males, with a mean age of 22 years (range 19–28 years). Of interest is that our current ongoing pilot case for the integrated clinical pathway is a young mixed-race female. Females are more likely to be undiagnosed, and clinical presentation can be different (Attoe & Climie 2023).

There are challenges with suspected ADHD and diagnostic assessment. In our Trust we have a stand-alone adult ADHD diagnostic assessment service for formal diagnosis, using DSM criteria with 3 phases of assessment. However, this service has significant referral rates, and due to capacity and resources, has a very long waiting list (18 months to 2 years).

As part of our quality improvement effort, we have collaboratively (with the adult ADHD service) worked towards an integrated pathway to improve efficiency and time to diagnostic assessment (reduce delays) for patients in EIP suspected with comorbid ADHD, with our first pilot case ongoing. We have developed a clinical pathway to aid clinicians in management of those with confirmed and suspected comorbid ADHD, to improve patient outcomes.

There are further training needs to effectively support and manage ADHD comorbidity in those with psychosis under the care of Early Intervention Psychosis.

